# Nitric oxide augments mesenchymal stem cell ability to repair liver fibrosis

**DOI:** 10.1186/1479-5876-10-75

**Published:** 2012-04-25

**Authors:** Gibran Ali, Sadia Mohsin, Mohsin Khan, Ghazanfar Ali Nasir, Sulaiman Shams, Shaheen N Khan, Sheikh Riazuddin

**Affiliations:** 1 National Center of Excellence in Molecular Biology, University of the Punjab, Lahore, Pakistan

**Keywords:** Liver fibrosis, Mesenchymal stem cells (MSCs), Hepatic stellate cells (HSCs), Nitric oxide

## Abstract

**Background:**

Liver fibrosis is a major health problem worldwide and poses a serious obstacle for cell based therapies. Mesenchymal stem cells (MSCs) are multipotent and important candidate cells for future clinical applications however success of MSC therapy depends upon their homing and survival in recipient organs. This study was designed to improve the repair potential of MSCs by transplanting them in sodium nitroprusside (SNP) pretreated mice with CCl_4_ induced liver fibrosis.

**Methods:**

SNP 100 mM, a nitric oxide (NO) donor, was administered twice a week for 4 weeks to CCl_4_-injured mice. MSCs were isolated from C57BL/6 wild type mice and transplanted in the left lateral lobe of the liver in experimental animals. After 4 weeks, animals were sacrificed and liver improvement was analyzed. Analysis of fibrosis by qRT-PCR and sirius red staining, homing, bilirubin and alkaline phosphatase (ALP) serum levels between different treatment groups were compared to control.

**Results:**

Liver histology demonstrated enhanced MSCs homing in SNP-MSCs group compared to MSCs group. The gene expression of fibrotic markers; αSMA, collagen 1α1, TIMP, NFκB and iNOS was down regulated while cytokeratin 18, albumin and eNOS was up-regulated in SNP-MSCs group. Combine treatment sequentially reduced fibrosis in SNP-MSCs treated liver compared to the other treatment groups. These results were also comparable with reduced serum levels of bilirubin and ALP observed in SNP-MSCs treated group.

**Conclusion:**

This study demonstrated that NO effectively augments MSC ability to repair liver fibrosis induced by CCl_4_ in mice and therefore is a better treatment regimen to reduce liver fibrosis.

## Background

Liver fibrosis is most often characterized by accumulation of wound healing myofibroblasts that replace normal hepatic tissue with scar at the site of injury [1,2]. Activated hepatic stellate cells (HSCs) produce high levels of extracellular matrix (ECM) proteins further contributing to scar development [3]. Liver transplantation is a suitable treatment modality, however, lack of available donors, immune rejection and overall cost of the procedure warrants new therapies for liver fibrosis [4]. There has been a great interest in therapeutic applications of bone marrow derived MSCs that have the potential to differentiate into hepatic lineages both in vitro and in vivo and can improve liver function [5-8]. Although recognized as a viable therapeutic option, MSC therapy has been surrounded by issues of poor cell viability and survival post transplantation [9] thereby supporting development of alternate treatment regimens.

One of the hallmarks of liver injury is the development of scar tissue as a consequence of HSC activation. HSCs are located within the space of Disse in liver sinusoids and comprise about 15% of total cell number in the liver [10]. In response to liver injury, HSCs switch from quiescent vitamin A storing cells to proliferative, α smooth muscle actin expressing cells, up regulating synthesis of ECM proteins [11]. There is 50–70 fold increase in the expression of type I collagen which is initiated and maintained by profibrotic cytokines like TGFβ family members and connective tissue growth factor (CTGF) [12,13]. Prolonged injury results in failure to respond to negative feedback regulation of collagen synthesis and deposition of cross-linked type I collagen fibrils, which are resistant to proteolytic degradation leading to alteration of the normal liver ECM and change in organ architecture [14].

Fibrogenic transformation of HSCs in response to liver damage has been proposed as a critical mechanism for liver failure. Removal of activated HSCs through apoptosis or programmed cell death can induce spontaneous liver recovery [15-17]. NO has been recently investigated as apoptotic inducer of activated HSCs but whether NO donors can be used in combination with MSC transplantation remains unknown [18-20]. In the present study, we demonstrate augmented MSC ability to repair fibrotic liver as a consequence of NO induced HSC apoptosis. Improvements mediated by this synergistic treatment of fibrotic liver with MSC and NO donor are evident structurally and functionally with enhanced homing of transplanted cells and significant reduction in fibrosis. These results validate the utility of NO induced HSC apoptosis as an effective way to enhance MSC potential for treatment of liver fibrosis.

## Materials and methods

### Animals

The investigation conforms to the *Guide for the Care and Use of Laboratory Animals* published by the US National Institutes of Health (NIH Publication No. 85–23, revised 1985). All animals were treated according to procedures approved by the Institutional Review Board (IRB) at the National Center of Excellence in Molecular Biology, Lahore, Pakistan.

### Cell isolation and culture

Bone marrow derived MSCs were isolated according to the procedure described previously [21]. MSCs were grown and sub-cultured till second passage and double labeled with PKH26 (Sigma Aldrich, USA) for the cell membrane and 4-6-diamidino-2- phenylindole (DAPI) for the nuclei (Sigma Aldrich, USA) according to the manufacturer’s instructions.

### Liver fibrosis model and sodium nitroprusside treatment

Female C57BL/6 mice aged 6–8 weeks and weighing 20–25 g were used in experiments. All animals were housed in conventional cages under controlled conditions of temperature (23 ± 3 C^o^) and relative humidity (50% ± 20%), with light illumination for 12 h/day. The animals were allowed access to food and water ad libitum throughout the experimental periods. To induce hepatic fibrosis, CCl_4_ (1 μl/g) was administered twice a week to animals as described previously [22]. After 4 weeks of CCl_4_ treatment, 100 mM SNP was dissolved in saline water (200 μl) and injected intraperitoneally to SNP and SNP-MSCs groups twice a week for 4 weeks. Griess reagent was prepared according to manufacturer’s instruction (Oxford biomedical research Inc, USA), was added to the serum samples and OD was measured at 540 nm with an ELISA plate reader. Serum concentration of total nitrite was measured 4 h after last SNP treatment. Mice were randomly divided (n = 11) into vehicle, CCl_4_, MSCs, SNP and SNP-MSCs groups. During drug administration period CCl_4_ injections were continued to all animals except to vehicle group.

### Cell transplantation

MSCs labeled with PKH26 were transplanted in MSCs and SNP-MSCs (n = 11) groups in concentration of 1 × 10^6^ cells/100 μl/animal at 2–3 different points directly in the left lateral lobe of the liver. Mice from SNP group were sham operated and received only PBS. All animals were kept under intensive care after operation. Animals received CCl_4_ injections once a week during post transplantation period until sacrificed after 4 weeks.

### Blood biochemistry

Blood samples were taken from all experimental groups (n = 11) at 4 weeks after cell transplantation. Serum was isolated and the amount of bilirubin (Diazyme Europe, Gmbh) and alkaline phosphatase (ALP) (Bioassay System, USA) was estimated using commercial kits according to the manufacturer’s protocol.

### Gene expression profiling

RNA from liver tissue of experimental groups was extracted using TRIZOL reagent (Invitrogen, Inc. USA). cDNA was synthesized using 1 μg of total RNA by cDNA synthesis kit (Fermentas). Gene Specific primers (Table 1) were designed using online software Primer3 (http://frodo.wi.mit.edu/primer3/). Analysis of real time RT-PCR gene expression (αSMA, collagen1α1, TIMP, NF-кB and albumin) in experimental groups (n = 3) was carried out using SYBR Green PCR Super Mix (BioRad Lab, CA, USA). The relative gene expression was then analyzed using SDS software (ABI). β-actin was used as an internal control.

**Table 1:** Primer sequences

**Primers**	**Forward reverse**
αSMA	CTGACAGAGGCACCACTGAA AGAGGCATAGAGGGACAGCA
Collagen1α1	GCCAAGAAGACATCCCTGAA GGCAGAAAGCACAGCACTC
TIMP	CATCTGGCATCCTCTTGTTG CTCGTTGATTTCTGGGGAAC
NFкB	GCACCTGTTCCAAAGAGCAC GTGGAGTGAGACATGGACACAC
Albumin	CGACTATCTCCAGCAAACTG GTCTCAGCAACAGGGATACA
β-Actin	ACTGCTCTGGCTCCTAGCAC ACATCTGCTGGAAGGTGGAC

### Histological analysis

Livers were isolated and fixed in 4% paraformaldehyde and paraffin embedded. Sections 5 μm thick were mounted on glass slides and 3 sections per animal and 3 animals per group were labeled with α-smooth muscle actin (α-SMA) (1:400; Sigma), Albumin (1:50; Abcam), cytokeratin-18 (1:50; Santa Cruz), eNOS (1:50; Santa Cruz) and iNOS (1:50; Santa Cruz) as primary antibodies while anti-mouse FITC, TRITC and peroxidase conjugated were used as secondary antibodies. MSCs were localized by tracking PKH26 labeled MSCs in CCl_4_ injured fibrotic liver tissue. Fluorescence images were taken by an Olympus BX-61 microscope loaded with DP 70 camera.

### Measurement of liver fibrosis

Fixed livers were embedded in paraffin and sections were cut from different lobes of the liver and Sirius red staining was done [22]. Images of the fibrotic area from 3 sections per animal and 3 animals per group were taken by an Olympus BX-61 microscope equipped with Digital Camera DP-70 (Olympus, Japan). Fibrosis and total area of each image was measured and the percentage of fibrotic area was calculated using Image J software.

### TUNEL

Apoptosis was measured using TUNEL assay in all experimental groups to analyze HSC death in response to SNP treatment as previously described [21].

### Statistical analysis

Quantitative data of 3 sections per animal and 3 animals per experimental group was obtained for sirius red staining and were expressed as ± SEM. Analysis for percentage of fibrosis area, bilirubin and ALP between different treatment groups vs control was performed by one-way ANOVA with bonferroni post-hoc test. P-value of less than 0.05 was considered statistically significant.

## Results

### Gene expression profiling

**Figure 1:**
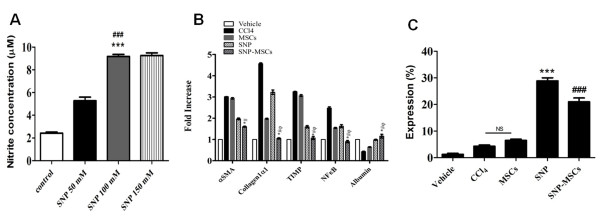
**Gene expression profiling after dose standardization of SNP.A**) Serum Nitrite concentration (μM). Values are mean ± SEM; n = 6. ^*^P < 0.05 for SNP 100 mM vs. control; ^#^P < 0.05 for SNP 100 mM vs. SNP 50 mM. **B**) mRNA expression after 4 weeks of treatment showed significantly reduced expression of α-SMA, collagen1α1, TIMP1 and NF-кB compared to CCl_4_ group. On the other hand, Albumin mRNA expression showed significant improvement after SNP-MSCs treatment. P-value < 0.05 was considered significant n = 3.^*^P < 0.05 for SNP-MSCs vs. CCl_4_; ^#^P < 0.05 for SNP-MSCs vs. MSCs; ^φ^P < 0.05 for SNP-MSCs vs. SNP. **C**) Expression (%) of SMA+/TUNEL + cells in all groups indicating HSC apoptosis after SNP administration. ^***^P < 0.001 for SNP vs. CCl_4_; ^###^P < 0.001 for SNP-MSCs vs. CCl_4_.

Dose optimization of SNP was determined prior to initiating experiments. Nitrite concentration in serum was significantly higher in mice treated with 100 mM as compared to 50 mM SNP and non treated group (Figure 1A). Interestingly, Increasing SNP concentration to 150 mM did not have a significant effect on the nitrite concentration. Therefore, 100 mM SNP was selected for further experiments. Gene expression profiling was conducted in experimental groups receiving SNP and MSCs in combination or alone. HSCs are believed to be the main ECM-producing cells in the liver [10] and express α-SMA. We observed a 3.0 fold increase in mRNA levels of α-SMA after treatment with CCl_4_ compared to 2.9 and 1.9 fold increase observed in MSCs and SNP treated groups. However, SNP-MSCs treatment resulted in only 1.6 fold increase in α-SMA which is significantly lower than other groups (Figure 1B). A similar pattern was observed in mRNA levels of collagen, TIMP and NF-кB, which are critical factors of liver fibrosis and were higher in the CCl_4_ group, decreased after MSCs or SNP treatment but SNP-MSC treatment further significantly reduced the expression of these markers. Conversely, Albumin level showed 1.2 fold increase in SNP-MSCs treatment compared to 0.9, 0.6 and 0.4 fold in SNP, MSCs and CCl_4_ groups respectively (Figure 1B). In addition, expression analysis of SMA, a marker for activated HSCs and TUNEL showed 28.8% SMA+/TUNEL + cells in SNP and 21.8% in SNP-MSCs groups (Figure 1C). Animals with CCl_4_ or MSCs treatment alone resulted in 4.3% and 6.5% SMA+/TUNEL + cells respectively.

### SNP-MSCs improve hepatic microenvironment

**Figure 2:**
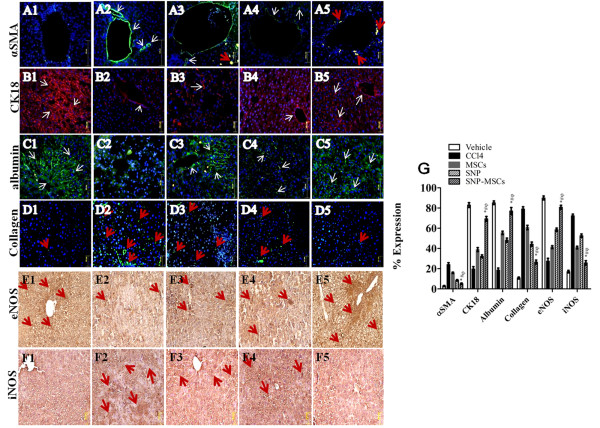
**Augmentation of hepatic microenvironment.** (**A**) SNP-MSCs resulted in the decreased expression of fibrotic marker (α-SMA), Collagen1 (**D**) and iNOS (**F**) and increased expression of hepatic markers cytokeratin-18 (**B**), albumin (**C**) and eNOS (E) in 1 = Vehicle, 2 = CCl_4_, 3 = MSCs, 4 = SNP, 5 = SNP-MSCs). G) Quantification of α-SMA, Collagen1, cytokeratin18, albumin, eNOS and iNOS in all treatment groups. All values are expressed as mean ± SEM. P-value < 0.05 was considered significant. ^*^P < 0.05 for SNP-MSCs vs. CCl_4_; ^#^P < 0.05 for SNP-MSCs vs. MSCs; ^φ^P < 0.05 for SNP-MSCs vs. SNP.

SNP-MSCs treatment resulted in reduced expression of αSMA and Collagen 1 (5.1%, 26.6%) compared to CCl_4_ injured liver (24.1%, 79.1%), MSCs (15.9%, 60.8%) and SNP (8.4%, 44.3%) groups (Figure 2 A, D & G) conferring to our real time PCR results (Figure 1B). Similarly expression of hepatic markers, cytokeratin-18 and albumin was increased significantly in SNP-MSCs group (69.3%, 77.1%) compared to CCl_4_ group (19.9%, 18.7%) and SNP alone (32.2%, 48.3%) or MSCs alone (38.9%, 55.4%) groups (Figure 2 B, C & G).

Acute liver injury is accompanied by inflammation and expression of NOS proteins. Among the two constitutive isoforms of NOS, eNOS expression (27.7%) was decreased in CCl_4_ injured liver, while the expression of inducible form iNOS (72.4%) was increased in the CCl_4_ injury (Figure 2 E-G). Increased iNOS or reduced eNOS have been shown to induce development of fibrosis in CCl_4_ liver injury [23] is in accordance with our results. SNP-MSCs treatment significantly increased the expression of eNOS (80.8%) while iNOS (25.9%) was significantly reduced compared to other treatment groups (Figure 2 E-G).

### SNP-MSCs reduce liver fibrosis

**Figure 3:**
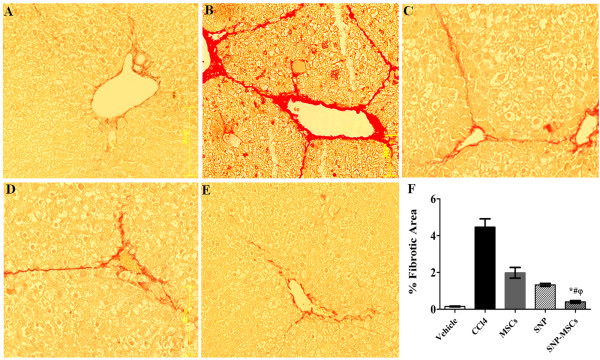
**Representative micrograph of hepatic tissue stained with Sirius red showing collagen deposition in various treatment groups.** (**A** = Vehicle, **B** = CCl_4_, **C** = MSCs, **D** = SNP, **E** = SNP-MSCs). **F**) Quantitative analysis of fibrosis in different experimental groups. One way ANOVA was applied to check the significance of the data. All values are expressed as mean ± SEM. P-value < 0.05 was considered significant. ^*^P < 0.05 for SNP-MSCs vs. CCl_4_; ^#^P < 0.05 for SNP-MSCs vs. MSCs; ^φ^P < 0.05 for SNP-MSCs vs. SNP.

SNP-MSCs treatment (Figure 3 E) resulted in significant reduction of liver fibrosis as measured by picrosirius procedure compared to MSCs alone, SNP alone, CCl_4_ groups and vehicle (Figure 3 A-D). Quantification of collagen fibers was done by image J software and showed marked increase in percentage of fibrotic area after CCl_4_ administration that was reduced significantly after SNP-MSCs treatment compared to other groups (Figure 3 F). Fibrotic area (%) was reduced significantly (0.4 ± 0.3) in SNP-MSCs group as compared to other treatment groups (CCl_4_ group = 4.4 ± 2.4, MSCs group = 1.9 ± 1.5 and SNP group = 1.3 ± 0.4).

### SNP-MSCs enhance homing and commitment

**Figure 4:**
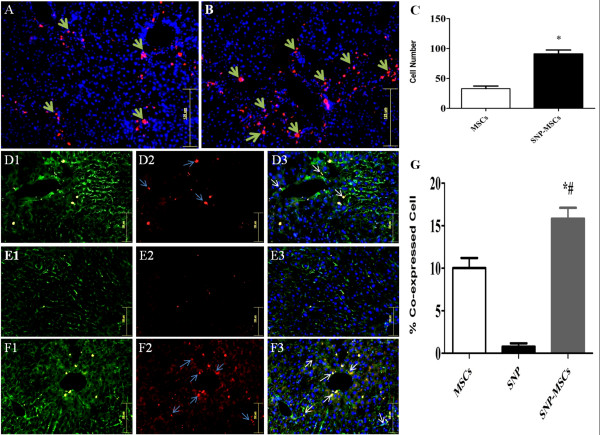
**MSCs homing and commitment.** Homing of transplanted cells in MSCs (**A**) and SNP-MSCs (**B**) groups. DAPI was used to identify nuclei. (Magnification = 200X). **C**) Quantification of engrafted cells in MSCs and SNP-MSCs groups. *p < 0.05 was considered to be significant. **F**) Significantly higher numbers of MSCs (white arrows) expressing albumin were present SNP-MSCs group as compared to other treatment groups. PKH26 (Red), Albumin (Green) and nuclei were counter stained with DAPI (blue). **G**) Quantification of engrafted cells in MSCs, SNP and SNP-MSCs groups. P-value < 0.05 was considered significant. ^*^P < 0.05 for SNP-MSCs vs. MSCs; ^#^P < 0.05 for SNP-MSCs vs. SNP.

Enhanced homing and localization of PKH-26/DAPI labeled MSCs was observed in CCl_4_ injured liver with engrafted cells observed in all lobes of the liver, indicating cell migration from left lateral lobe to other injury sites (Figure 4 A-B). However SNP-MSCs treated animals showed better homing ability than non SNP treated MSCs group with significant increase in the number of cells observed (Figure 4 C).

When co-expression was monitored, there is increased expression of albumin with higher number of PKH26 positive cells (16 cells/field) in SNP-MSCs group as compared to MSCs group (10 cells/field) (Figure 4 D-G).

### Functional recovery after SNP-MSCs

**Figure 5:**
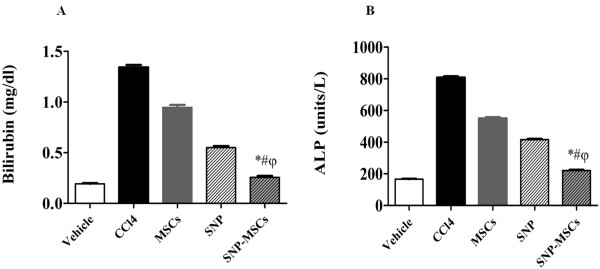
**Functional analysis after SNP-MSCs treatment:** (**A**) Bilirubin (**B**) Alkaline phosphatase (ALP) levels in different experimental groups. One way ANOVA was used to test the significance of the data among groups (n = 11). P-value < 0.05 was considered significant. ^*^P < 0.05 for SNP-MSCs vs. CCl_4_; ^#^P < 0.05 for SNP-MSCs vs. MSCs; ^φ^P < 0.05 for SNP-MSCs vs. SNP.

To further evaluate the role of SNP-MSCs in preventing hepto-cellular injury, we measured the serum concentrations of bilirubin and ALP from different treatment groups. At week 4, the serum bilirubin level in the SNP-MSCs group was 0.2 mg/dl, which were significantly lower than those in CCl_4_ (1.3 mg/dl), SNP (0.5 mg/dl) and MSCs alone (0.9 mg/dl) groups (Figure 5 A). Similarly, the serum ALP levels in the SNP-MSCs group were (220 units/L), which were significantly lower than those in CCl_4_ (810 units/L), SNP (420 units/L) and MSCs alone (550 units/L) groups (Figure 5 B). Collectively, these results indicate a superior ability of SNP-MSCs to augment hepatic function compared to either of the treatments alone or the CCl_4_ treatment group.

## Discussion

Hepatic injury is most often characterized by widespread hepatocyte damage leading to fibrosis and scar development. Injured hepatocytes and their metabolites activate kupffer cells [24,25] releasing cytokines such as transforming growth factor-α (TGF-α), platelet-derived growth factor (PDGF) and tumor necrosis factor-α (TNF-α) [3]. These factors activate HSCs which upon stimulation lose retinoid storing ability and transform into myofibroblasts [26,27]. Transformation of HSCs from their quiescent state to a fibrotic cell critically affects hepatic repair and may also impact the outcome of cell based therapeutic options. Bone marrow derived MSCs have the ability to repair damaged liver [28,29] however, extensive fibrosis, scar development and lack of survival may influence regeneration ability. Strategies targeting removal of activated HSCs have shown to reduce fibrosis and augment liver function [30,31]. A combined approach aimed at specific removal of activated HSCs would improve hepatic milieu allowing MSCs to survive, engraft and differentiate into hepatocytes. Therefore, in the present study we employed NO for induction of HSC apoptosis in combination with MSC transplantation in mouse model for liver fibrosis. Our intended hypothesis was to probe the combined effect of NO and MSCs as each treatment alone has been shown to be protective against hepatic injury [18,19,32,33].

Follistatin and Gliotoxin like compounds have been tested for their utility to induce apoptosis of HSCs but in an unspecific manner [16,34]. Many studies have reported that high levels of NO induce apoptosis in many cell types primarily by the effect of peroxynitrite that increases mitochondrial permeability [35,36]. NO donors can exert an antifibrogenic action as NO has negative regulatory properties specifically on activated HSCs migration, contraction and proliferation in fibrotic liver [19,37,38]. Therefore, we expected that the effect of NO administration exogenously would be beneficial in reducing liver fibrosis. Several studies have shown therapeutic effects of MSCs in liver disease [39-41] but, controversial observations still exist [42,43]. Studies have shown that bone marrow derived stem cells can be a source of collagen and contribute to liver damage [27,42]. Therefore, improvement in liver environment is essential for the successful outcome of MSC therapy. Apoptosis of activated HSCs combined with transplantation of MSCs would be able to recover hepatic microenvironment yielding better results. A previous study reported improvement in liver fibrosis with combined treatment of FGF2 and MSCs by regulating the expression of metalloproteinases (MMPs) and ultimately reduction in matrix proteins [44].

Activated HSCs express α-SMA in the periportal and perisinusoidal areas [45] and that can be indicative of prevalent liver fibrosis [46-49]. Our results showed increased levels of α-SMA in CCl_4_ injured liver indicating possible HSC activation while combined treatment with SNP-MSCs resulted in significant reduction in α-SMA mRNA level (Figure 1). In addition, a significant reduction in other indicators of liver fibrosis such as collagen 1α1, TIMP and NF-κB showed decrease in mRNA level after SNP-MSCs administration compared to CCl_4_ treated group or single treatment of SNP or MSCs (Figure 1). Treatment with SNP-MSCs resulted in significant increase in albumin, a hepatocyte marker compared to CCl_4_ group.

Immunohistochemical analysis further corroborated the real time PCR results indicating significant reduction in levels of α-SMA and iNOS concurrent with increased cytokeratin-18, albumin and eNOS after treatment with SNP-MSCs at 4 weeks compared to CCl_4_ group and both single treatment groups (Figure 2). Similarly, Sirius red staining demonstrated significant decline in fibrotic area after treatment with SNP-MSCs after 4 weeks compared to CCl_4_ group providing evidence of considerable augmentation of hepatic microenvironment and reduction in fibrosis (Figure 3).

Improved hepatic microenvironment as evidenced by attenuated fibrosis resulted in significant increase in the number of transplanted cells in damaged liver of SNP-MSCs group compared to MSCs only group (Figure 4) coinciding with previous findings demonstrating direct homing of MSCs to injured liver [22]. In addition, increased MSCs differentiation was observed in SNP-MSCs group compared to MSCs only group as evidenced by levels of albumin (Figure 4). Significant reduction in the bilirubin and ALP serum levels was observed in experimental animals transplanted with MSCs and pretreated with SNP compared to CCl_4_ group and SNP or MSCs alone treatment groups (Figure 5). Bilirubin and ALP serum levels have been used previously in various studies [14,22,50] as indicators of improved liver function, thereby meriting use of both these parameters.

Autologous stem cell therapy represents an attractive treatment modality for liver fibrosis however; extensive fibrosis and scar formation can limit efficacy of the therapy. Activated HSCs play a critical role in mediating liver fibrosis significantly contributing towards the prognosis of the disease. Transplantation of MSCs together with NO pretreatment of the injured liver tissue represents a novel and promising strategy to augment the repair ability of stem cells in hepatic fibrosis. Furthermore, MSCs transplantation in NO pretreated injured liver tissue demonstrates better survival, differentiation and functional abilities. These findings establish an efficient way to enhance MSC ability to repair liver fibrosis by targeting HSC apoptosis through administration of NO.

## Conclusion

We have demonstrated that nitric oxide treatment can significantly improve the ability of MSCs to repair liver fibrosis. Nitric oxide induces apoptosis of activated HSCs which are considered to be one of the critical mediators of liver fibrosis. Activation of HSCs in the fibrotic liver transforms these vitamin A storing cells into fibroblasts. We have used nitric oxide treatment of the fibrotic liver to induce HSC apoptosis thereby improving liver microenvironment. MSCs transplantation in fibrotic livers treated with nitric oxide results in reduction of fibrosis, augmentation of liver function and improved MSCs survival compared to livers only receiving MSCs. Therefore, we report here a clinically viable treatment modality combining nitric oxide treatment with MSC transplantation for the treatment of liver fibrosis.

## Competing interests

The authors have no financial conflicts of interest.

## Authors’ contributions

GA and SM designed research; GA, GAN, SS performed research; GA and SM analyzed data; and GA, MK, SNK and SM wrote the paper. All authors read and approved the final manuscript.
